# Nomogram constructed by immunological and inflammatory indicators for predicting prognosis of patients with esophageal squamous cell carcinoma treated with neoadjuvant chemoradiotherapy plus surgery

**DOI:** 10.3389/fonc.2022.882900

**Published:** 2022-07-29

**Authors:** Yun Luo, Xue-Fen Weng, Jia-Tao Huang, Xue-Hao Hu, Lai-Feng Wei, Yi-Wei Lin, Tian-Yan Ding, Biao Zhang, Ling-Yu Chu, Can-Tong Liu, Yu-Hui Peng, Yi-Wei Xu, Fang-Cai Wu

**Affiliations:** ^1^ Department of Clinical Laboratory Medicine, The Cancer Hospital of Shantou University Medical College, Shantou, China; ^2^ Precision Medicine Research Center, Shantou University Medical College, Shantou, China; ^3^ Research Center for Advanced Optics and Photoelectronics, Department of Physics, College of Science, Shantou University, Shantou, China; ^4^ Shantou Center, Guangdong Esophageal Cancer Institute, Guangzhou, China; ^5^ Department of Radiation Oncology, The Cancer Hospital of Shantou University Medical College, Shantou, China

**Keywords:** esophageal squamous cell carcinoma, prognosis, IgA, CRP, neoadjuvant chemoradiotherapy

## Abstract

**Objectives:**

At present, esophageal squamous cell carcinoma (ESCC) patients accepting neoadjuvant chemoradiotherapy (nCRT) plus surgery lack corresponding prognostic indicators. This study aimed to construct a prognostic prediction model for ESCC patients undergoing nCRT and surgery based on immune and inflammation-related indicators.

**Methods:**

We retrospectively analyzed the levels of serum immune- and inflammation-related indicators of ESCC patients before receiving nCRT plus surgery in the training cohort (99 patients) and validation cohort (67 patients), which were collected from 2007 to 2020. Univariate and multivariate Cox survival analyses were conducted to evaluate the indicators to set up a nomogram associated with the patients’ overall survival (OS). The prediction accuracy and discriminative ability of the nomogram were measured by the concordance index (C-index), decision curve, calibration curve, integrated discrimination improvement (IDI), and net reclassification improvement (NRI).

**Results:**

Univariate and multivariate Cox analyses demonstrated that immune globin A (IgA) and C-reactive protein (CRP) were independent risk factors. A nomogram based on IgA, CRP, and cTNM stage was established for predicted OS in the training cohort and validated in the validation cohort. The C-index of the nomogram was 0.820 (95% CI: 0.705–0.934), which was higher than that of the cTNM stage (0.655 (95% CI: 0.546–0.764), *p* < 0.05) in the training cohort, and similar results were observed in the validation cohort (0.832 (95% CI: 0.760–0.903 vs 0.635 (95% CI: 0.509–0.757), *p* < 0.001). Furthermore, the prediction accuracy and net benefit of the nomogram verified by the calibration curve, decision curve, NRI, and IDI were satisfactory in the training and validation cohorts.

**Conclusion:**

The newly constructed nomogram concluding serum IgA, CRP, and cTNM stage might be helpful in the prognosis prediction for ESCC patients receiving nCRT plus surgery.

## Introduction

Esophageal cancer is the seventh most common malignant cancer all over the world and the sixth leading cause of cancer mortality (1). In 2018, 508,585 esophageal cancer-related deaths occurred globally (2). Approximately 90% of the pathological types of esophageal cancer are esophageal squamous cell carcinoma (ESCC) (3, 4). For the treatment of ESCC, traditional curative esophagectomy was a major selection. However, in patients with locally advanced esophageal cancer (T_3–4a_N_0–1_M_0_), only radical resection is accompanied by a high recurrence rate and mortality rate of 3- to 5-year overall survival (OS) (5, 6). Therefore, combined modality therapy is necessary. Some studies indicated that when compared with preoperative chemotherapy or surgery alone in patients with locoregional esophageal cancer, neoadjuvant chemoradiotherapy was connected with improved OS and disease-free survival (DFS) (7–11). Several markers (such as Rad51, osteopontin, plasma fibrinogen, and soluble interleukin-6 receptor) have also been reported to be associated with the prognosis of ESCC patients who received preoperative chemoradiotherapy (12–16). However, the treatment effect and prognostic indicator before neoadjuvant chemoradiotherapy still need further research.

This is a broad consensus that TNM staging is pivotal and irreplaceable for the diagnosis and treatment of tumors including esophageal carcinoma. Clinical studies indicated that the TNM stage is an independent prognostic factor for OS (17, 18). However, the classification of the cTNM stage mainly depends on computed tomography (CT) and endoscopic ultrasonography (EUS). Studies reported that the sensitivity of EUS (usually less than 50% sensitivity) for detecting metastatic nodes was low. The specificity to discriminate the N0 from a node-positive disease of CT was just 38.7%, and CT was unreliable for local staging (19–21). Based on these findings, only depending on a single cTNM stage may lead to deviations in the accuracy of prediction when establishing a prognostic model for ESCC patients with neoadjuvant chemoradiotherapy (nCRT) plus surgery. Therefore, it is necessary to construct a multivariate prognostic model.

It is increasingly recognized that cancer-related inflammation (CRI) is related to tumor progression. Inflammation in tumor microenvironments can promote cell proliferation and inhibit the adaptive immune response (22). Systemic inflammation score (SIS) has played an independent role in the prognosis of ESCC patients (23). In cancer-related immunology, immune cells exerted anti-tumor capacity through different mechanisms such as binding ligands on the surface of tumor cells through self-surface receptors; meanwhile, pro-inflammatory and immunomodulatory cytokines such as IL-12, TNF-α, IFN-γ, and IL-6 were released during the process. These cytokines would cause changes in other inflammatory substances such as C-reactive protein (CRP). In the cancer immunoediting process, antibodies are involved in the event of antibody-dependent cell-mediated cytotoxicity. Moreover, the level of different antibodies was variable in tumor location or blood (22, 24). The connection between cancer-related inflammation and immunology is tight. The antibody, inflammatory factor, and cytokine generated the re-sculpting of the tumor immune and inflammatory microenvironment (25). However, the relationship between serum immune and inflammation indicators and prognosis with ESCC patients who received nCRT plus surgery is indistinct. The target of the study was to construct a nomogram constructed with immune and inflammatory indicators. Through this nomogram, we can macroscopically recognize the benefit that ESCC patients could obtain after nCRT plus surgery.

## Methods and materials

### Study population

In the study, 166 ESCC patients who were treated with nCRT followed by esophagectomy in the Cancer Hospital of Shantou University Medical College were enrolled from 2007 to 2020. A total of 99 patients were included in the training cohort from 2007 to 2020, and 67 patients were included in the validation cohort from 2013 to 2019. All cases were diagnosed as esophageal squamous cell carcinoma by pathological biopsy. Pathological and clinical data were collected through case history records. Basic information includes weight, height, age, gender, body mass index (BMI), and tumor location. Hematology indexes included IgA, IgG, IgM, CRP, CRP/albumin (CRP/ALB), complement 3 (C3), C4, lymphocyte ratio (LY%), lymphocytes count (LY#), monocyte ratio (MO%), monocyte count (MO#), neutrophil ratio (NE%), and neutrophil count (NE#). In order to obtain a consistent tumor cTNM stage, the depth of tumor invasion (T), lymph node metastasis (N), and distant organ metastasis (M) were recorded by the examination results of computed tomography and endoscopic ultrasound. The cTNM stage was assessed according to the standards of the American Joint Committee on Cancer (AJCC) Staging Manual of Esophageal Cancer (8th edition) (26). Patients who had a history of other cancers or suffered from inflammatory diseases such as chronic gastritis, inflammatory bowel disease, autoimmune diseases, and infectious diseases, which may influence the levels of the above-mentioned pretreatment serum markers, were excluded.

The main regimen of chemotherapy was cisplatin combined with paclitaxel, vinorelbine, docetaxel, or fluorouracil. Depending on the patient’s condition, at least two cycles of chemotherapy were conducted. Concurrent radiotherapy began on the first day of chemotherapy. The gross tumor volume included the primary tumor and enlarged regional lymph nodes. The total planned dose for the planning target volume was 40–50 Gy in 20–25 fractions within 5 weeks. Esophagectomy was performed 4 to 6 weeks after the completion of nCRT.

The time from the date of diagnosis to any form of death or the last follow-up was defined as OS. The study was allowed by the ethics committee of the Cancer Hospital of Shantou University Medical College.

### Construction and evaluation of nomogram for prognosis prediction

Categorical variables were adopted for all continuous variables in both cohorts, according to the best cutoff values determined by the X-tile program (27). Combined with independent prognostic factors determined by multiple Cox regression analysis, a nomogram was established to predict 1-year and 3-year OS of ESCC patients in the training cohort, and the prediction accuracy and applicability of the nomogram were verified in the validation cohort. The discrimination of the nomogram was assessed by the concordance index (C-index). The accuracy and benefits of the new model were assessed by integrated discrimination improvement (IDI), net reclassification improvement (NRI), and decision curve analysis (DCA). The goodness-of-fit model was determined by the calibration curve. Based on the nomogram, the total points were calculated, and the survival curve was plotted for risk stratification.

### Statistical analysis

IBM SPSS statistical software was used for statistical analysis, version 19.0 (IBM Corp., Chicago, IL), and the R software, version 4.0.4, for Windows. R packages including ggplot2, ggpubr, survminer, survival, rms, pec, magrittr, dplyr, survIDINRI, and Hmisc were used in the analysis. Included indicators of a significant level of *p* ≤ 0.05 in the univariate Cox analysis were used in the multivariate Cox analysis. In the multivariate Cox analysis, indicators with a significant level of *p* ≤ 0.05 were determined as an independent prognostic factor. The Kaplan–Meier curve was carried out to obtain the survival rate, and the log-rank test was used to test it.

## Results

### Characteristics of patients

**Table 1** shows the clinical characteristics of ESCC patients receiving nCRT plus surgery in the training and validation cohorts. The cutoff values of continuous variables were as follows: age (64 years), IgA (3 g/L), IgG (12.9 g/L), IgM (0.9 g/L), CRP (4.4 mg/L), CRP/ALB (0.1), C3 (1.3 g/L), BMI (19.6), C4 (0.3 g/L), LY% (23.9), LY# (1.7 * 10^9^/L), MO% (8.0), MO# (0.6 * 10^9^/L), NE% (66.1), and NE# (5.1 * 10^9^/L). The cutoff values in the validation cohort were consistent with the training cohort.

**Table 1:** Patient demographics and clinical characteristics.

Characteristic	Training cohort		Validation cohort	
	No	%	No	%
Gender				
Female	20	20.2	13	19.4
Male	79	79.8	54	80.6
Age (years)				
<64	73	73.7	55	82.1
≥64	26	26.3	12	17.9
Clinical TNM stage				
II–III	55	55.6	38	56.7
IV	44	44.4	29	43.3
Location				
Up	25	25.3	13	19.4
Middle	63	63.6	45	67.2
Low	11	11.1	9	13.4
BMI				
<19.6	32	32.3	23	34.3
≥19.6	67	67.7	44	65.7
IgG (g/L)				
<12.9	62	62.6	36	53.7
≥12.9	37	37.4	31	46.3
IgA (g/L)				
<3	75	75.8	50	74.6
≥3	24	24.2	17	25.4
IgM (g/L)				
<0.9	16	16.2	3	4.5
≥0.9	83	83.8	64	95.5
CRP (mg/L)				
<4.4	80	80.8	49	73.1
≥4.4	19	19.2	18	26.9
CRP/ALB				
<0.1	75	75.8	47	70.1
≥0.1	24	24.2	20	29.9
C3 (g/L)				
<1.3	50	50.5	59	88.1
≥1.3	49	49.5	8	11.9
C4 (g/L)				
<0.3	42	42.4	51	76.1
≥0.3	57	57.6	16	23.9
LY%				
<23.9	51	51.5	33	49.3
≥23.9	48	48.5	34	50.7
LY# (10^9^/L)				
<1.7	38	38.4	23	34.3
≥1.7	61	61.6	44	65.7
MO%				
<8.0	57	57.6	43	64.2
≥8.0	42	42.4	24	35.8
MO# (10^9^/L)				
<0.6	56	56.6	38	56.7
≥0.6	43	43.4	29	43.3
NE%				
<66.1	55	55.6	38	56.7
≥66.1	44	44.4	29	43.3
NE# (10^9^/L)				
<5.1	55	55.6	40	59.7
≥5.1	44	44.4	27	40.3

TNM, tumor node metastasis; IgG, immune globin G; IgA, immune globin A; IgM, immune globin M; CRP, C-reactive protein; ALB, albumin; C3, complement 3; C4, complement 4; BMI, body mass index; LY%, lymphocyte ratio; LY#, absolute count of lymphocytes; MO%, monocyte ratio; MO#, absolute count of monocytes; NE%, neutrophil ratio; NE#, absolute count of neutrophils.

### Univariate and multivariate Cox analyses of overall survival

**Figure 1:**
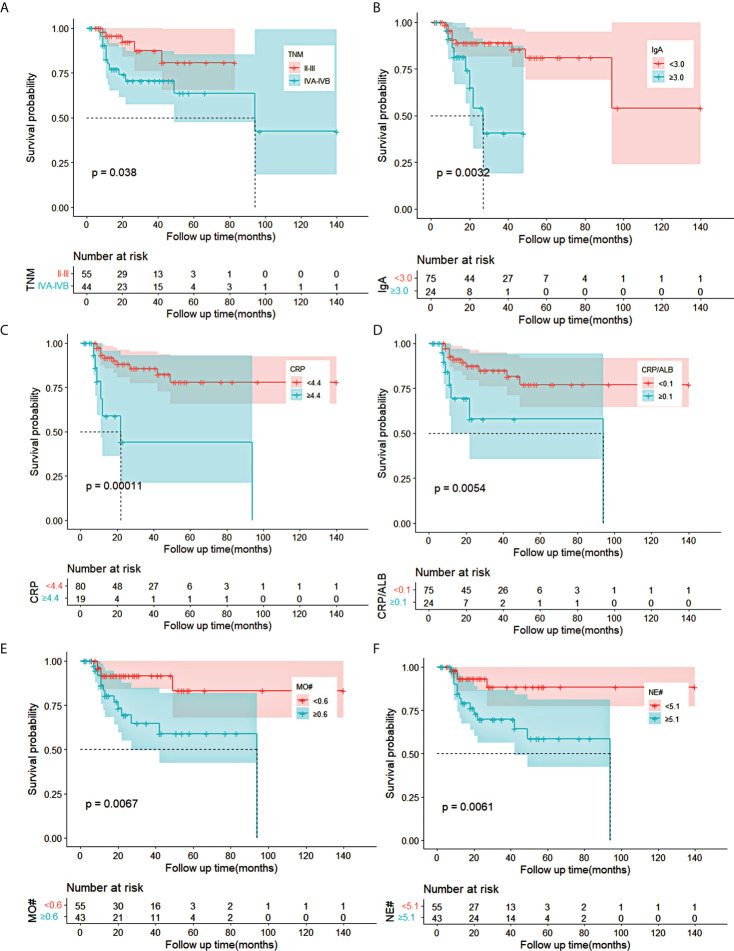
Kaplan–Meier curves for OS in ESCC patients receiving nCRT plus surgery in training cohort. **(A–F)** The survival curves of clinical TNM stage, IgA, CRP, CRP/ALB, MO#, and NE# in ESCC patients. OS, overall survival; ESCC, esophageal squamous cell carcinoma; nCRT, neoadjuvant chemoradiotherapy; IgA, immune globin A; CRP, C-reactive protein; ALB, albumin; MO#, absolute count of monocytes; NE#, absolute count of neutrophils.

Univariate analysis demonstrated that the cTNM stage (*p* = 0.049), IgA (*p* = 0.007), CRP (*p* = 0.001), CRP/ALB (*p* = 0.009), MO# (*p* = 0.011), and NE# (*p* = 0.014) were statistically significant for OS in the training cohort. Based on the cTNM stage, CRP, CRP/ALB, IgA, MO#, and NE#, the Kaplan–Meier curves of OS were significantly different through the log-rank test (*p* < 0.05) as shown in **Figure 1**. The collinearity diagnosis of these six variables suggested that collinearity existed between CRP and CRP/ALB. After the multivariate Cox analysis by the forward stepwise method, which was adopted to exclude the variables with collinearity, the IgA (*p* = 0.017, hazard ratio (HR) = 3.498, 95% CI: 1.255–9.748) and CRP (*p* = 0.002, HR = 4.936, 95% CI: 1.828–13.330) were evaluated as independent factors for OS in the training cohort (**Table 2**).

**Table 2:** Univariate and multivariate Cox analyses of training cohort for OS.

Characteristic	Univariate analysis			Multivariate analysis		
	HR	95% CI	*p*	HR	95% CI	*p*
Gender						
Female	Reference					
Male	2.387	0.548–10.406	0.247			
Age (years)						
<64	Reference					
≥64	1.116	0.397–3.136	0.835			
Clinical TNM stage						
II–III	Reference					
IV	2.852	1.003–8.107	0.049			
Location						
Up	Reference					
Middle	0.966	0.305–3.055	0.952			
Low	1.814	0.401–8.214	0.440			
BMI						
<19.6	Reference					
≥19.6	0.577	0.228–1.458	0.245			
IgG						
<12.9	Reference					
≥12.9	0.465	0.152–1.428	0.181			
IgA						
<3	Reference			3.498	1.255–9.748	0.017
≥3	4.060	1.477–11.164	0.007			
IgM						
<0.9	Reference					
≥0.9	0.602	0.211–1.713	0.341			
CRP						
<4.4	Reference			4.936	1.828–13.330	0.002
≥4.4	5.686	2.125–15.213	0.001			
CRP/ALB						
<0.1	Reference					
≥0.1	3.624	1.372–9.575	0.009			
C3						
<1.3	Reference					
≥1.3	1.205	0.464–3.131	0.702			
C4						
<0.3	Reference					
≥0.3	2.517	0.931–6.808	0.069			
LY%						
<23.9	Reference					
≥23.9	0.627	0.242–1.622	0.335			
LY#						
<1.7	Reference					
≥1.7	1.964	0.694–5.557	0.204			
MO%						
<8.0	Reference					
≥8.0	2.000	0.761–5.255	0.160			
MO#						
<0.6	Reference					
≥0.6	3.837	1.367–10.771	0.011			
NE%						
<66.1	Reference					
≥66.1	1.503	0.590–3.828	0.393			
NE#						
<5.1	Reference					
≥5.1	4.044	1.327–12.328	0.014			

HR, hazard ratio; 95% CI, 95% confidence interval; TNM, tumor node metastasis; IgG, immune globin G; IgA, immune globin A; IgM, immune globin M; CRP, C-reactive protein; ALB, albumin; C3, complement 3; C4, complement 4; BMI, body mass index; LY%, lymphocyte ratio; LY#, absolute count of lymphocytes; MO%, monocyte ratio; MO#, absolute count of monocytes; NE%, neutrophil ratio; NE#, absolute count of neutrophils; OS, overall survival.

### Nomogram construction for the prediction of overall survival

**Figure 2:**
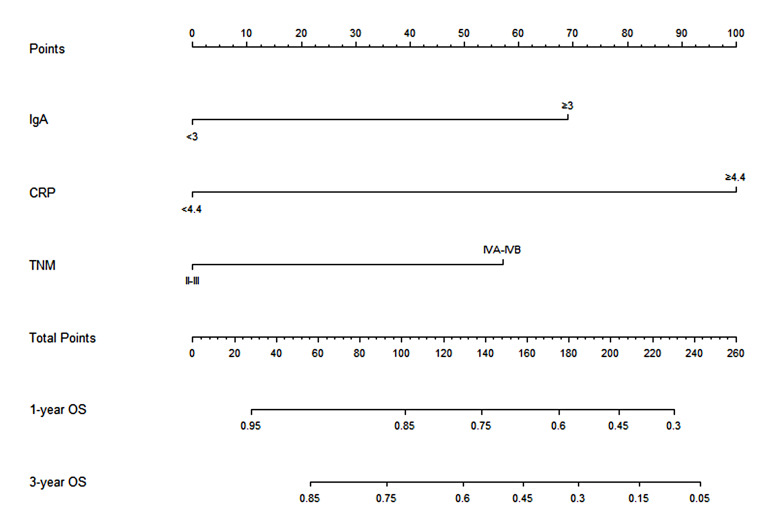
Nomogram based on CRP, IgA, and clinical TNM stage for predicting the 1- and 3-year OS in ESCC patients who received nCRT plus surgery, by summing up the points identified on the points scale for each variable. The total points projected on the bottom scales determined the probability of 1- and 3-year survival. CRP, C-reactive protein; IgA, immune globin A; OS, overall survival; ESCC, esophageal squamous cell carcinoma; nCRT, neoadjuvant chemoradiotherapy.

Through the multivariate Cox analysis, CRP and IgA were identified to meaningfully influence the OS. Meanwhile, the cTNM stage is a significant factor in the evaluation of cancer treatment decision-making and prognosis before any cancer-related therapy. Thus, the cTNM stage was included in the nomogram model. Hence, the nomogram was established using the cTNM stage, CRP, and IgA to forecast the 1- and 3-year OS in the training cohort **Figure 2**). In the nomogram, CRP had a greater effect on OS as compared with IgA or the cTNM stage. Risk points related to different levels of each predictive indicator were obtained by drawing a straight line to the “Points” line according to the corresponding indicator value and then summing up these risk points to obtain total points. A straight line was painted down from the total point to the straight line of 1- and 3-year OS, and the intersection point is the 1- or 3-year survival rate.

### Prediction accuracy and net benefit for nomogram

**Figure 3:**
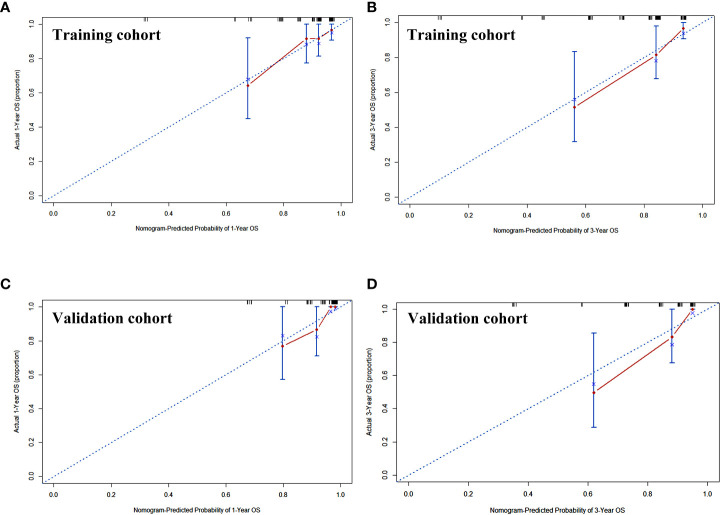
The calibration curve of the nomogram to predict the overall survival rate of 1 and 3 years in training cohort **(A, B)** and validation cohort **(C, D)**. x-Axis was the nomogram-predicted probability of 1 or 3 years of OS. y-Axis was the actual OS of the patients included in the study. OS, overall survival.

**Figure 4:**
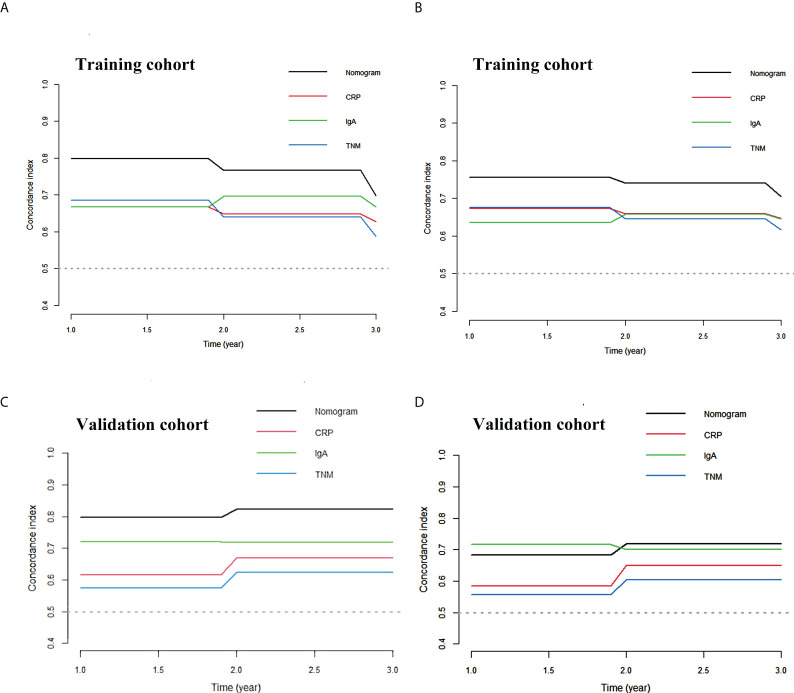
The C-index curve of the nomogram. **(A)** The C-index curve under the time distribution of 3 years to predict OS in training cohort. **(B)** The internal verification by Bootstrap algorithm of 3 years to predict OS in training cohort. **(C)** The C-index curve under the time distribution of 3 years to predict OS in validation cohort. **(D)** The internal verification by Bootstrap algorithm of 3 years to predict OS in validation cohort. OS, overall survival.

Calibration curves of this nomogram to predict the 1- and 3-year OS were drawn in both cohorts (**Figure 3**). The calibration curve demonstrated that the predicted OS based on the nomogram excellently fitted the actual OS in either the training cohort or the validation cohort. Moreover, the C-index indicating prediction accuracy of the nomogram was 0.820 (95% CI: 0.705–0.934), higher than that of the cTNM stage, CRP, and IgA (0.655 (95% CI: 0.546–0.764), 0.656 (95% CI: 0.540–0.773), and 0.624 (95% CI: 0.501–0.747), respectively, *p* < 0.05) in the training cohort. Similar results were identified in the validation cohort, with the C-index of 0.832 (95% CI: 0.760–0.903), better than those of the cTNM stage, CRP, and IgA (0.635 (95% CI: 0.509–0.757), 0.679 (95% CI: 0.557–0.792), and 0.678 (95% CI: 0.549–0.800)) (**Table 3**). The C-index curves under the time distribution of 3 years to predict OS of ESCC patients who received nCRT plus surgery in the training and validation cohorts are shown in **Figures 4A, C**, and the C-index of the nomogram was contrasted with every single variable. In **Figures 4B, D**, the internal verification by the Bootstrap algorithm was calculated to obtain a more reliable C-index in both cohorts. Both of them revealed that the C-index of the nomogram was good.

**Table 3:** The C-index of CRP, IgA, clinical TNM stage, and nomogram for prediction of OS.

Factors	Training cohort		Validation cohort	
	C-index (95% CI)	*p*	C-index (95% CI)	*p*
For OS				
IgA	0.624 (0.501–0.747)		0.678 (0.549–0.800)	
CRP	0.656 (0.540–0.773)		0.679 (0.557–0.792)	
cTNM stage	0.655 (0.546–0.764)		0.635 (0.509–0.757)	
Nomogram	0.820 (0.705–0.934)		0.832 (0.760–0.903)	
Nomogram vs IgA		0.0147		0.0084
Nomogram vs CRP		0.0467		0.0785
Nomogram vs cTNM stage		0.0493		<0.001

p-values are calculated based on normal approximation using function rcorrp.cens in Hmisc package. Nomogram: IgA+ CRP+ clinical TNM stage.

OS, overall survival; C-index, concordance index; 95% CI, 95% confidence interval; IgA, immune globin A; CRP, C-reactive protein.

**Figure 5:**
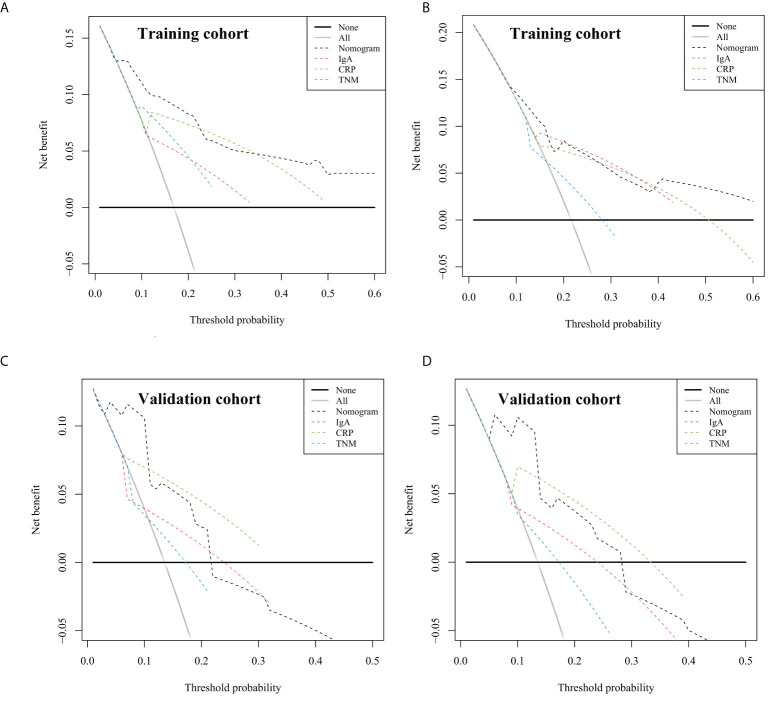
Decision curve analysis of the nomogram for overall survival (OS), compared with CRP, IgA, and clinical TNM stage. **(A)** The decision curve for 1-year OS in training cohort. **(B)** The decision curve for 3-year OS in training cohort. **(C)** The decision curve for 1-year OS in validation cohort. **(D)** The decision curve for 3-year OS in validation cohort. The straight black line represents the assumption that all patients die, and the horizontal line represents the assumption that no deaths happened. CRP, C-reactive protein; IgA, immune globin A.

Furthermore, decision curve analysis was applied to evaluate the net benefit of the nomogram in both cohorts. As shown in **Figure 5**, the nomogram has a higher net benefit than that of other indicators to predict overall survival rates of 1 and 3 years. Moreover, as shown in **Table 4**, the NRI demonstrated that the prediction accuracy of the nomogram was better than CRP, IgA, and cTNM stage (NRI > 0), and the IDI suggested that the accuracy of the nomogram to predict 1- and 3-year OS was increased by 8.7% and 23.2%, respectively, as compared with the cTNM stage in the training cohort. Similarly, the NRI and IDI revealed that the prediction accuracy of the nomogram was better than IgA and the cTNM stage. To conclude, the prediction accuracy and net benefit of nomogram were higher than those of other assessment systems, which were identified through calibration curve, C-index, DCA, IDI, and NRI in the training cohort. Moreover, the prediction accuracy and applicability of the nomogram were verified in the validation cohort. Therefore, the model was reliable.

**Table 4:** Predictive improvement of the nomogram for the training and validation cohorts.

	1 year				3 years			
	NRI%	*p*	IDI%	*p*	NRI%	*p*	IDI%	*p*
Training cohort								
Nomogram vs IgA	27.6	0.040	11.1	0.034	9.6	0.182	10.6	0.060
Nomogram vs CRP	1.9	0.304	2.6	0.484	26.7	0.058	10.8	0.050
Nomogram vs cTNM stage	35.5	0.072	8.7	0.074	65.6	0.004	23.2	0.000
Validation cohort								
Nomogram vs IgA	53.6	0.070	2.2	0.470	61.3	0.012	4.5	0.320
Nomogram vs CRP	−38.8	0.484	−0.1	1.269	−42.1	0.236	−0.2	0.795
Nomogram vs cTNM stage	45.4	0.034	3.7	0.322	63.5	0.010	5.4	0.308

NRI, net reclassification improvement; IDI, integrated discrimination improvement; IgA, immune globin A; CRP, C-reactive protein.

### Risk stratification based on nomogram

**Figure 6:**
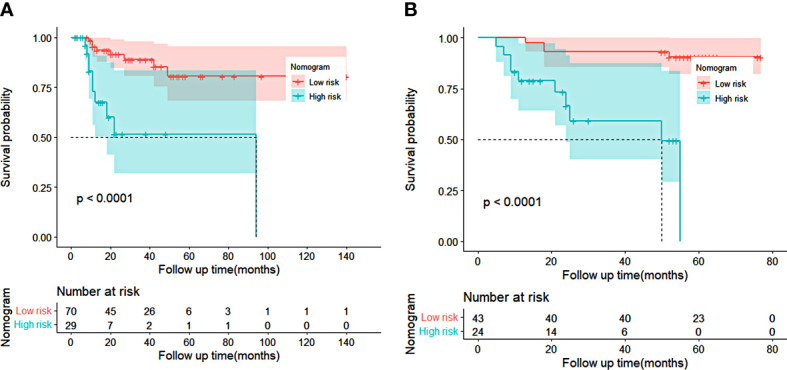
Kaplan–Meier curves of risk stratification for overall survival (OS) based on the predictions of the nomogram in training cohort **(A)** and validation cohort **(B)**. Low risk: total points <100 for OS. High risk: total points ≥100 for OS.

Then we calculated the predicted total points according to the established nomogram in both cohorts, utilized the X-tile program to obtain the best cutoff value (100 for OS), and subdivided patients into the low-risk or high-risk groups according to the cutoff value. We used Kaplan–Meier survival analysis to evaluate survival. As indicated in **Figure 6**, the OS of the high-risk group was shorter than that of the low-risk group in both cohorts (*p* < 0.05). This revealed that the nomogram was feasible for risk stratification and OS prediction for patients before receiving nCRT plus surgery.

## Discussion

Esophageal cancer as the sixth leading cause of cancer death causes huge economic pressure, especially for developing countries, and many of the tumors will have progressed to advanced stages during the diagnosis. Moreover, the benefit rate of surgery alone in advanced patients was low (1, 3, 4). Neoadjuvant chemoradiation is recommended for advanced esophageal cancer patients. Chemotherapy medicines such as carboplatin, paclitaxel, cisplatin, and fluorouracil were frequently selected in combination therapy with radiation, and the pathological complete response and overall survival rate were improved as compared with surgery alone (28, 29). Nevertheless, the benefit rate of nCRT was closely related to the individual. Therefore, it was necessary to establish a prognostic model for ESCC patients who received nCRT. As research reported, the DFS was shorter for the patients with preoperative hyperfibrinogenemia (fibrinogen > 350 mg/dl), and the plasma fibrinogen level was a biomarker for predicting postoperative recurrence (14). Although some prognostic indicators were confirmed as independent risk factors for OS or DFS, most of them were single indicators. This meant the predicted bias may exist for prognosis. Further, the significance of the model based on combined indicators deserved to evaluate. Here, we established the nomogram model according to the preoperative cTNM stage, serum CRP, and IgA for predicting the prognosis of ESCC patients receiving nCRT plus surgery and verified the nomogram in the validation cohort. Our nomogram showed improved prediction accuracy for prognosis, compared with the cTNM stage.

The connection between cancer-related inflammation and protective tumor immunity is dynamic. Consequently, evaluating the immune and inflammation status of tumor patients was of positive significance for prognosis. CRP, which existed in the form of pentamer in plasma, was synthesized mainly by hepatocytes (30–33). As an acute reaction protein, under normal circumstances, the content of CRP in human serum was low, but the CRP concentration even increased 1,000-fold in response to inflammation or tissue damage (34). Several studies reported that the high level of CRP as an independent prognostic factor was connected with OS in gastric, lung, ovarian, and esophageal cancers (35–40), and the high preoperative or postoperative CRP levels were related to worse survival prognosis in ESCC patients (35, 40–42). These results were consistent with the finding of serum CRP observed in our study. Survival analysis showed that a high CRP level (≥4.4 mg/L) before nCRT plus surgery was associated with worse OS. In our nomogram, CRP had a strong effect on predicting OS. IgA, which was produced by plasma cells, existed in the mucous membrane, tissues, and blood. Furthermore, IgA was the second most abundant immunoglobulin isotype in serum. IgA drove passive immunity-related functions. Recently, IgA-induced inflammation in diseases has been discussed (43, 44). Furthermore, as reported by Shalapour et al., IgA+ cells induced by an inflammatory environment dismantled anti-liver tumor immunity (45). IgA, which effectuated an important role in the diagnosis of chronic lymphocytic leukemia (CLL), was an independent prognostic factor for disease progression, survival, and infection in CLL (46). However, studies about the prognostic prediction ability of IgA on solid tumors were insufficient. In our work, we revealed that IgA was an independent risk factor for OS of ESCC patients who received nCRT plus surgery, and survival analysis revealed that high IgA level (≥3.0 g/L) was related to worse 1- or 3-year OS.

In our study, the prediction accuracy of the nomogram was satisfactory, as the C-index of the nomogram adopting internal verification through the Bootstrap algorithm was 0.820 (95% CI: 0.705–0.934) in the training cohort and 0.832 (95% CI: 0.760–0.903) in the validation cohort, which was higher than those of any other single indicators including the cTNM stage. DCA, IDI, NRI, and calibration curve consistently certified that the net benefit, model fit, and accuracy of nomogram were satisfactory in both cohorts. These consequences confirmed that the prognostic prediction of the nomogram established with the levels of immunity and inflammation-related indicators was reliable.

However, the shortcomings of our study still existed. Firstly, the sample size was relatively small in our study. This may cause a bias in the results. For example, the TNM stage was generally used to evaluate cancer patient survival in clinical practice. However, in our study, the cTNM stage was statistically related to patient survival, but not an independent prognostic factor in multivariate Cox analysis. This is mainly attributed to the small sample size and the majority of enrolled patients with advanced stage. In the future, expanding the samples is important to address this issue. Secondly, both the training cohort and the validation cohort were from the same hospital. Arrangement of the verification in external cohorts was required in the future. Thirdly, the retrospective nature of analyses was another limitation of this work. Thus, further research with prospective design is warranted to validate our constructed nomogram.

## Conclusion

In our study, we established a nomogram based on serum IgA, CRP, and cTNM stage to predict the prognosis of ESCC patients receiving nCRT plus surgery. The predicted ability, accuracy, and applicability were satisfactory, which were verified in a validation cohort.

## Data availability statement

The raw data supporting the conclusions of this article will be made available by the authors, without undue reservation.

## Ethics statement

The studies involving human participants were reviewed and approved by the Hospital Ethics Committee in the Cancer Hospital of Shantou University Medical College. The patients/participants provided their written informed consent to participate in this study. Written informed consent was obtained from the individual(s) for the publication of any potentially identifiable images or data included in this article.

## Author contributions

YL and X-FW designed the study, collected and analyzed the patient samples and clinical data, and wrote the manuscript. J-TH and X-HH collected patient samples and clinical data, analyzed and interpreted the clinical data, and revised the manuscript. L-FW, Y-WL, and T-YD collected patient samples and clinical data, analyzed the data, and revised the manuscript. BZ, L-YC, and C-TL collected patient samples and clinical data, analyzed the clinical data, and revised the manuscript. Y-HP, Y-WX, and F-CW conceptualized and designed the study, supervised the project, and revised the manuscript. All authors contributed to the article and approved the submitted version.

## Funding

This work was funded by grants from Science and Technology Planning Project of Shantou City (No. 220506106490650), the Natural Science Foundation of China (No. 81972801), Guangdong Basic and Applied Basic Research Foundation (No. 2019A1515011873), and 2020 Li Ka Shing Foundation Cross-Disciplinary Research Grant (No. 2020LKSFG01B).

## Acknowledgments

The authors thank the Department of Clinical Laboratory Medicine of the Cancer Hospital of Shantou University Medical College for their contributions to data collection and analysis.

## Conflict of interest

The authors declare that the research was conducted in the absence of any commercial or financial relationships that could be construed as a potential conflict of interest.

## Publisher’s note

All claims expressed in this article are solely those of the authors and do not necessarily represent those of their affiliated organizations, or those of the publisher, the editors and the reviewers. Any product that may be evaluated in this article, or claim that may be made by its manufacturer, is not guaranteed or endorsed by the publisher.
